# Lead poisoning outbreak among opium users in the Islamic Republic of Iran, 2016–2017

**DOI:** 10.2471/BLT.17.196287

**Published:** 2018-02-05

**Authors:** Talat Ghane, Nasim Zamani, Hossein Hassanian-Moghaddam, Ali Beyrami, Alireza Noroozi

**Affiliations:** a Drug and Poison Information Centre, Food and Drug Administration of the Islamic Republic of Iran, Tehran, Iran.; b Social Determinants of Health Research Center, Shahid Beheshti University of Medical Sciences, Teheran, Islamic Republic of Iran.; c Department of Clinical Toxicology, School of Medicine, Shahid Beheshti University of Medical Sciences, Arabi Ave, Daneshjoo Blvd, Velenjak, Tehran 19839-63113, Islamic Republic of Iran.; d Office of Narcotics and Controlled Substances, Food and Drug Administration of the Islamic Republic of Iran, Tehran, Islamic Republic of Iran.; e Iranian National Center for Addiction Studies, Tehran University of Medical Sciences, Tehran, Islamic Republic of Iran.

## Abstract

**Objective:**

To describe an outbreak of lead poisoning among opium users in the Islamic Republic of Iran and estimate the number of affected people in the country.

**Methods:**

We used data from the country’s largest poison treatment centre to illustrate the epidemiology of an outbreak of lead poisoning in oral opium users. We describe the government’s referral and treatment guidelines in response to the outbreak. Based on the number of individuals treated and previous studies on the prevalence of oral opium use we estimated the total number of people at risk of lead-contaminated opium nationwide.

**Findings:**

In February 2016, we noticed a steep increase in the numbers of oral opium users referred to our poison treatment centre with abdominal pain, anaemia and constipation. Numbers peaked in June 2016 but the outbreak was ongoing in August 2017. The mean blood lead level in a sample of 80 patients was 140.3 µg/dL (standard deviation: 122.6). Analysis of an illegal opium sample showed 3.55 mg lead in 1 g opium. Treatment was exposure reduction with opioid substitutes and laxatives, or chelation therapy if indicated. Over 7 months, 4294 poison cases were seen at main referral hospitals in Tehran out of an estimated 31 914 oral opium users in the city. We estimate more than 260 000 out of 773 800 users nationwide remain untreated and at risk of poisoning.

**Conclusion:**

Lead-contaminated opium and heroin that has transited through the Iranian markets is a global risk and highlights a need for better monitoring of illegal drug supplies.

## Résumé

**Objectif:**

Décrire la vague d'intoxications au plomb survenue chez les consommateurs d'opium en République islamique d'Iran et estimer le nombre de personnes concernées dans le pays.

**Méthodes:**

Nous avons utilisé les données du plus grand centre antipoison du pays pour illustrer l'épidémiologie d'une vague d'intoxications au plomb survenue chez les consommateurs d'opium par voie orale. Nous décrivons les directives du gouvernement en matière de traitement et d'orientation vers des services spécialisés suite à cette flambée. À partir du nombre de personnes traitées et de précédentes études sur la prévalence de la consommation d'opium par voie orale, nous avons estimé le nombre total de personnes susceptibles de consommer de l'opium contaminé au plomb dans le pays.

**Résultats:**

En février 2016, nous avons constaté une forte augmentation du nombre de consommateurs d'opium par voie orale orientés vers notre centre antipoison avec des douleurs abdominales, une anémie et une constipation. Cette hausse a culminé au mois de juin 2016 mais la vague se poursuivait en août 2017. La plombémie moyenne, sur un échantillon de 80 patients, était de 140,3 µg/dL (écart type: 122,6). L'analyse d'un échantillon d'opium illicite a révélé la présence de 3,55 mg de plomb dans 1 g d'opium. Le traitement a consisté à réduire l'exposition et à prendre des substituts aux opiacés et des laxatifs, ou des agents chélateurs lorsque cela était indiqué. Sur 7 mois, 4294 personnes intoxiquées ont été reçues dans les principaux hôpitaux centraux de Téhéran, sur un total estimé de 31 914 consommateurs d'opium par voie orale dans la ville. Nous estimons que plus de 260 000 consommateurs sur 773 800 dans le pays n'ont, à ce jour, pas reçu de traitement et courent un risque d'intoxication.

**Conclusion:**

L'opium contaminé au plomb et l'héroïne qui ont transité sur les marchés iraniens représentent un risque mondial et mettent en avant la nécessité de mieux contrôler l'offre de drogues illicites.

## Resumen

**Objetivo:**

Describir un brote de intoxicación con plomo entre consumidores de opio en la República Islámica del Irán y estimar la cantidad de personas afectadas en el país.

**Métodos:**

Utilizamos datos del centro de tratamiento de intoxicaciones más grande del país para ilustrar la epidemiología de un brote de intoxicación con plomo en consumidores de opio por vía oral. Describimos la referencia y orientaciones de tratamiento del gobierno en respuesta al brote. Basado en el número de individuos tratados y estudios anteriores sobre la prevalencia del uso de opio por vía oral, estimamos el número total de personas en riesgo por el opio contaminado con plomo a nivel nacional.

**Resultados:**

En febrero de 2016, advertimos un aumento pronunciado en los números de consumidores de opio por vía oral remitidos a nuestro centro de tratamiento de intoxicación con dolor abdominal, anemia y constipación. Los números tuvieron un pico en junio de 2016 pero el brote fue continuo en agosto de 2017. La media del nivel de plomo en sangre en una muestra de 80 pacientes fue 140.3 µg/dL (desviación estándar: 122.6). El análisis de una muestra ilegal de opio mostró 3.55 mg de plomo en 1 g de opio. El tratamiento fue la reducción de la exposición con sustitutos de opio y laxantes, o terapia de quelación si se indicó. Durante 7 meses, 4294 casos de intoxicación se atendieron en hospitales principales de referencia en Teherán de un estimado de 31 914 consumidores de opio por vía oral en la ciudad. Estimamos que más de 260 000 de 773 800 consumidores en toda la nación continúan sin tratamiento y en riesgo de intoxicación.

**Conclusión:**

El opio y la heroína contaminados con plomo, que transitó a través de los mercados iraníes, es un riesgo global y destaca la necesidad de un mejor control de los suministros de droga ilegal.

## ملخص

**الغرض:**

وصف تفشي الإصابة بالتسمم بالرصاص بين متعاطي الأفيون في جمهورية إيران الإسلامية وتقدير عدد المصابين في البلاد.

**الطريقة:**

لقد استخدمنا بيانات من أكبر مركز لعلاج التسمم في البلاد لتوضيح الانتشار الوبائي لتفشي الإصابة بتسمم الرصاص بين متعاطي الأفيون عن طريق الفم. ونحن نصف المبادئ التوجيهية التي تتبعها الحكومة لتقديم العلاج وتحويل المرضى في إطار التصدي لتفشي الإصابة. وبناءً على عدد الأفراد الذين تم علاجهم والدراسات السابقة حول انتشار تعاطي الأفيون عن طريق الفم، فقد قمنا بتقدير العدد الإجمالي للأشخاص المعرضين لخطر الإصابة بالتسمم بفعل الأفيون الملوث بالرصاص على مستوى البلاد.

**النتائج:**

في شهر فبراير/شباط عام 2016، لاحظنا ارتفاعًا حادًا في أعداد من يتعاطون الأفيون عن طريق الفم الذين تمت إحالتهم إلى مركز علاج التسمم الخاص بنا وكانوا يعانون من آلام في البطن وفقر الدم والإمساك. في شهر يونيو/حزيران عام 2016 بلغت الأعداد ذروتها ولكن استمر التفشي في شهر أغسطس/آب عام 2017. وكان متوسط مستوي الرصاص في الدم المقاس في عينة مكونة من 80 مريضًا 140.3 ميكروجرام/ديسيلتر (الانحراف المعياري: 122.6). وقد أظهر التحليل لعينة من الأفيون غير الشرعي وجود 3.55 ميكروجرام من الرصاص في كل 1 جرام من الأفيون. كان العلاج يتم من خلال تخفيض وجود الأفيون باستخدام البدائل الأفيونية والملينات، أو عملية استخلاب (إزالة المعادن) عند الاقتضاء. على مدى 7 أشهر، شوهدت 4294 حالة تسمم في مستشفيات الإحالة الرئيسية في طهران من بين ما يقدر بنحو 31914 من متعاطي الأفيون عن طريق الفم في المدينة. وتشير تقديراتنا إلى وجود ما يزيد عن 260000 من بين 773800 متعاط على مستوى البلد بأكملها لا يزالون بلا علاج ومعرضين لخطر الإصابة بالتسمم.

**الاستنتاج:**

يمثل الهيروين والأفيون المسممان بالرصاص الذي انتقل عبر الأسواق الإيرانية خطرًا عالميًا، ويلقي الضوء على الحاجة إلى رقابة أفضل لإمدادات العقاقير غير الشرعية.

## 摘要

**目标:**

描述伊朗伊斯兰共和国鸦片使用者的铅中毒疫情，并估算该国的受影响人数。

**方法:**

使用该国最大的中毒治疗中心的数据来说明口服鸦片使用者铅中毒暴发的流行病学特征。我们描述了政府应对疫情的转诊和治疗指南。根据接受治疗的个体数量和之前关于口服鸦片使用率的研究，我们估计了全国范围内受到含铅鸦片影响的总人数。

**结果:**

2016 年 2 月，我们注意到转诊至中毒治疗中心的口服鸦片患者人数剧增，并伴有腹痛、贫血和便秘的症状。该数值在 2016 年 6 月达到峰值，但 2017 年 8 月时，疫情仍旧持续。80 例患者中，其中一个样本的血铅平均值为 140.3 µg/dL（标准偏差：122.6）。对某非法鸦片样本的分析显示，1 克鸦片的含铅量为 3.55 毫克。治疗方案为使用阿片类药物替代品和缓泻剂以降低接触影响，或者如有说明，则使用螯合疗法。7 个多月以来，德黑兰的主要转诊医院看诊了 4294 例中毒病例，据估计，该市约有 31 914 位口服鸦片使用者。我们预估全国范围内有 773800 位鸦片使用者中，其中超过 260000 位未接受治疗，有中毒风险。

**结论:**

通过伊朗市场流转的含铅鸦片和海洛因是一项全球性风险，突出了对更好地监管非法药物供应的需求。

## Резюме

**Цель:**

Описать вспышку отравления свинцом среди потребителей опиума в Исламской Республике Иран и оценить количество пострадавших людей в стране.

**Методы:**

Мы использовали данные, полученные от крупнейшего в стране токсикологического центра, чтобы проиллюстрировать эпидемиологию вспышки отравления свинцом у потребителей опиума пероральным путем. Мы приводим описание рекомендаций правительства в отношении направления и лечения в условиях вспышки. Основываясь на количестве лиц, получивших медицинскую помощь, и предыдущих исследованиях распространенности перорального потребления опиума, мы подсчитали общее число людей по всей стране, которые входят в группу риска отравления опиумом, загрязненным свинцом.

**Результаты:**

В феврале 2016 года мы заметили резкое увеличение числа потребителей опиума пероральным путем, поступивших в наш токсикологический центр с болью в животе, анемией и запорами. Пик их количества пришелся на июнь 2016 года, но в августе 2017 года вспышка еще продолжалась. Средний уровень свинца в крови у 80 пациентов составил 140,3 мкг/дл (стандартное отклонение: 122,6). Анализ образца незаконного опиума выявил содержание свинца, равное 3,55 мг в 1 г опиума. В качестве лечения для снижения воздействия использовались опиоиды-заместители и слабительные или при необходимости терапия хелатами. За 7 месяцев в основных больницах Тегерана было зарегистрировано 4294 случая отравления из приблизительно 31 914 потребителей опиума пероральным путем. По нашим оценкам, более 260 000 из 773 800 потребителей по всей стране остаются без медицинской помощи и подвергаются риску отравления.

**Вывод:**

Загрязненные свинцом опиум и героин, которые проходят через иранские рынки, представляют собой глобальный риск и указывают на необходимость лучшего мониторинга незаконных поставок наркотиков.

## Introduction

Substance abuse is an important social and public health problem in the Islamic Republic of Iran. The country has a long and porous border with Afghanistan, the main producer of illicit opium and heroin in the world.^1^ Opium and opium residue are the most commonly used drugs. According to a national household survey in 2011 an estimated 1 325 000 of the 53 million population aged 15‒65 years had used any illicit substance over the previous 12 months.^2^ Most them (1 181 900) were opium users,^2^ giving a 12-month prevalence of opium use disorder (criteria of the *Diagnostic and statistical manual of mental disorders*, fifth edition) of 2.23% (95% confidence interval: 1.83–2.62%). In 2015, the Iranian Drug Control Headquarters estimated that 2.8 million of 15‒64-year-olds were dependent on illicit drugs.^3^ The average age of opioid dependence was 32 years, with the average age of initiation of drug use in the early 20s. High numbers of road traffic crashes^4^ and crimes related to substance abuse^5^ have been linked to substance use in the country.

In response to the high burden of opioid use, the government adopted a treatment and harm reduction policy in 2002. A major concern was preventing a shift in the trend of substance abuse from oral opium to intravenous heroin and hence reducing the risk of injection-related transmission of diseases such as human immunodeficiency virus.^6^ Under this policy, an outpatient treatment programme was established and scaled up rapidly. Treatment with the opioid agonist drugs methadone, buprenorphine and opium tincture are available through a large network of outpatient clinics across the country.^7^ In 2017, more than 7000 methadone maintenance treatment clinics were providing treatment to 500 000 opioid-dependent people.

Opioids and pharmaceutical products have become the main causes of poisoning in the country. Each year more than 3000 citizens, mostly men, die due to substance overdose.^8^ Almost 1605 deaths due to acute substance use were reported by the Iranian Legal Medicine Organization (equivalent to the chief medical examiner or coroner) between April and September 2016, a 9.1% rise compared with the same time period in 2015 when there were 1471 deaths.^8^

A drug and poison information centre was first established in the capital city Tehran in 1995 and there are now 36 such centres nationwide.^9^^,^^10^ The centres provide information to the public and health-care providers, mostly via a telephone helpline, about use of medications, drug interactions and drug toxicity. Almost all hospital emergency departments treat poisoning cases and some have specialized clinical toxicology departments that are equipped with antidotes and provide specialized care for poisoned patients.^10^

Starting in February 2016 we noticed an increase in the numbers of opium users referred to our university hospital poison centre with severe abdominal pain, anaemia and constipation. Lead toxicity among opium users was already a concern in the country^11^^,^^12^ and we suspected the symptoms were due to lead contamination of illegal opium supplies. We consulted our department of clinical toxicology and started an investigation for the Iranian Ministry of Health in March 2016. The aims of the current study were to describe the outbreak of lead poisoning in opium users in the Islamic Republic of Iran and to estimate the numbers of affected people nationwide.

## Methods

In this observational study we describe the general features of the national outbreak of lead poisoning in opium users. We outline the health ministry response to the outbreak, including the referral and treatment strategy for suspected cases. We obtained data from the Iranian Food and Drug Administration on the annual amounts of opioid substitution medications distributed from 2010 to 2016 and on the national stocks of lead-chelation medications and the numbers of patients treated with them during the outbreak.

We used data from our poison treatment centre to illustrate the epidemiology of the outbreak. We extracted data from the hospital’s monthly administrative records and analysed the total numbers of patients referred with symptoms of lead poisoning to our centre over the period February 2016 to August 2017 and the numbers treated in outpatient clinics or admitted to the wards. We tested the blood lead level of all our patients, but mostly at follow-up not on presentation, as we did not have the capacity to test all patients on arrival. For this study, we selected a sample of 80 patients to illustrate the blood lead levels. Other laboratory examinations were complete blood count including haemoglobin level. We also used atomic absorption spectroscopy to measure the lead concentration in a sample of illegal opium obtained from a patient.

A suspected case of lead toxicity was defined as a patient with one of the following: (i) history of lead exposure as well as chronic abdominal colic, pallor or lead line (bluish pigmentation of the gingiva); (ii) history of lead exposure and two of the following clinical manifestations: prolonged constipation and neurological symptoms including weakness, headache, impatience, drowsiness, agitation or irritability; or (iii) presence of three of the following clinical manifestations: chronic abdominal colic, pallor, lead line, prolonged constipation, neurological symptoms including weakness, headache, impatience, drowsiness, agitation or irritability. History of lead exposure was defined as employment in certain high-risk industries or oral opium use. A probable case was defined as any suspected case with possible history of lead exposure with no definitive diagnosis. A confirmed case was a patient who met the probable case definition and had laboratory-confirmed lead toxicity (blood lead level ≥ 10 µg/dL in adults) in the emergency department or after discharge.^13^

Based on the number of confirmed cases of lead poisoning seen in the two main referral centres in Tehran and previous studies on the prevalence of opium use, we estimated the total number of number of people at risk of lead-contaminated opium in Tehran and nationwide.

## Results

### Outbreak and response

Within a month after the start of the outbreak in February 2016, physicians anecdotally reported thousands of opium users attending emergency departments throughout the country with severe abdominal pain not responding to any opioid-mimicking substance. Other findings included anaemia, constipation, seizures, loss of consciousness, weakness, muscle and bone pain, nausea and vomiting, ataxia, mutism, wrist drop, paraesthesia, encephalopathy and delirium.^14^^–^^17^ Initially diagnosed as acute abdominal emergencies, many of the patients underwent unnecessary surgery.^18^ Blood samples of admitted patients showed elevated lead levels and also random tests in samples of illegal opium obtained from patients showed high levels of lead.

Alerted to a possible epidemic of lead poisoning among opium users in Tehran and Kerman provinces, the health ministry developed guidelines on the diagnosis and referral of suspected cases. A one-day meeting in April 2016 was held to brief senior officers of all the Iranian medical universities, who supervise the health facilities in their area, about implementation of the guidelines. The guidelines were widely distributed to all emergency departments of public and private hospitals.^13^

### Case study

**Fig. 1:**
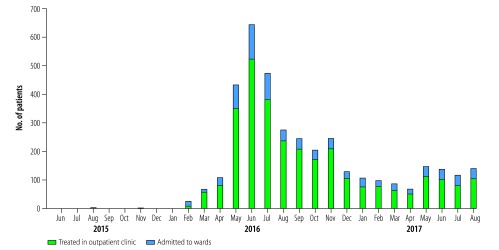
Number of patients treated for confirmed lead poisoning at Loghman-Hakim hospital poison centre during the outbreak in the Islamic Republic of Iran, June 2015 to August 2017

Loghman-Hakim hospital poison centre in Tehran is the largest referral centre for clinical toxicology in the country.^19^^,^^20^ On 14 February 2016 a patient who was reported to be a user of oral opium was admitted to the centre with abdominal pain, anaemia and constipation. A blood test showed a blood lead level of 137 µg/dL, considerably higher than the reference level of 10 µg/dL in adults.^21^ The patient had no history of occupational exposure to lead. A further 25 patients with suspected lead poisoning were referred to our centre that month. All patients reported being opium dependent, confirmed by our observations of opioid withdrawal syndrome during hospital treatment. The number of cases continued to rise in March and April, with a steep increase in May 2016, peaking in June 2016 at 645 cases (123 admitted to hospital, 522 treated as outpatients; Fig. 1). A slight increase in numbers was seen in May 2017 and the outbreak was ongoing in August 2017 with 141 cases referred. In a sample of 80 hospitalized patients we found a mean blood lead level of 140.3 µg/dL (standard deviation: 122.6; range: 47.3–1124 µg/dL). Analysis of the lead content of an illegal opium sample obtained from a patient showed 3.55 mg lead in 1 g opium. All patients were re-evaluated 1 month after discharge from hospital. Patients’ recovery was evaluated by asking about their signs and symptoms. The blood lead level was re-checked and patients who were symptom-free and had a lead level <  30 µg/dL were considered to require no further treatment except follow-up and advice on avoiding lead exposure.

### Treatment

**Fig. 2:**
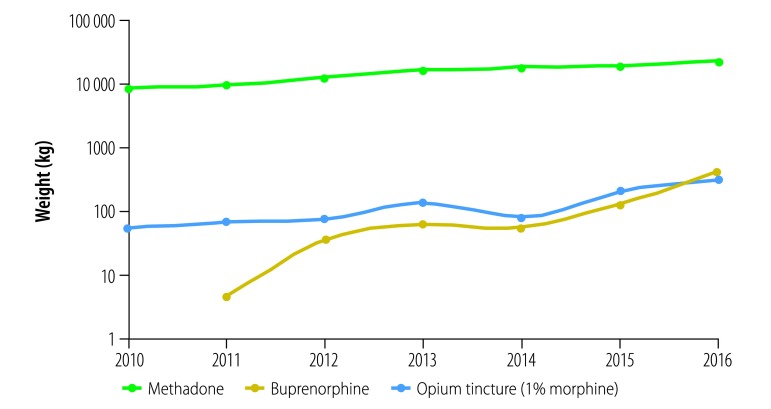
Trend in annual amounts of oral opioid medications distributed to treatment centres for opioid substitution therapy in the Islamic Republic of Iran, 2010–2016

Notes: *y*-axis data are the annual total weight of active ingredients distributed (logarithmic scale). Buprenorphine was distributed in 0.4, 2 or 8 mg tablets and buprenorphine/naloxone sublingual tablets of 2/0.5 and 8/2 mg. Methadone were distributed in 5, 20 or 40 mg tablets and syrups containing 5 mg/mL. Opium tincture was an alcoholic solution of 10 mg/mL opium, equivalent to 1 mg/mL morphine. Data were obtained from the Office of Narcotics and Controlled Substances, Food and Drug Administration of the Islamic Republic of Iran.

The first step in the government treatment recommendations was exposure reduction and decontamination. The health ministry used a media campaign via television, radio and mobile phone internet messaging and advice from staff at health centres to encourage substance users to withdraw illegal opium consumption and refer to outpatient substance treatment clinics. Despite the advice to cease opium ingestion completely, treatment centres used opioid substitution drugs methadone, buprenorphine, buprenorphine with naloxone and opium tincture (alcoholic solution of 10 mg/mL opium, equivalent to 1 mg/mL morphine) to provide a safe substitute for the contaminated opium. To respond to the increased demand for maintenance therapy, the ministry also distributed thousands of bottles of opium tincture to treatment centres who registered their requirements for opioids. Fig. 2 shows the annual amounts of opioid medications (weight of active ingredients) distributed in the whole country during the year of the outbreak (2016) compared with previous years (2010‒2015). Opium tincture requests increased from 125 kg in 2015 to 399 kg in 2016. Methadone and buprenorphine distribution in 2015 increased from 18 669 and 202 kg to 22 108 and 318 kg in 2016, respectively, a steady increase since 2012.

Treatment centres used laxative drugs such as polyethylene glycol, sorbitol and lactulose to accelerate defecation. Constipation following chronic opium use facilitates absorption of ingested lead in daily opium use. Using laxatives could therefore safely decrease lead absorption from the gastrointestinal tract and improve the abdominal pain often experienced by these patients. Antioxidants such as N-acetylcysteine, vitamin C and E, calcium and iron supplements and dairy products were also given to improve malnutrition and anaemia. 

Poisoned patients whose signs and symptoms had not resolved after discontinuing opium consumption or those who had not aware of their illnesses were referred for treatment. The recommendation was that lead-chelation medications dimercaprol, succimer, dimercaptosuccinic acid or sodium calcium edetate be administered at treatment centres to symptomatic patients with a blood lead level ≥ 40 µg/dL (Table 1). There were limited national stocks of chelation agents and the health ministry had to import further supplies on multiple occasions. The only other available drug was D-pencillamine, which is not the treatment of choice for lead poisoning because its efficacy is doubtful compared with the first-choice treatments and the long-term effects have not yet been evaluated.^22^ Succimer, sodium calcium edetate and dimercaprol were kept available in limited amounts to be used for severe cases. Fortunately, most patients responded to penicillamine in the early stages of poisoning and other treatment centres in the country have used the drug with good results.^15^ A total of 19 960 patients nationwide were given chelation therapy (Table 1).

**Table 1:** Lead-chelating medications distributed to treatment centres during the outbreak of lead poisoning among opium users in the Islamic Republic of Iran, February 2016 to August 2017

Drug, dose	Formulation	No. of doses available	Average daily dose, mg	Duration of treatment, days	No. of doses per course	Estimated no. of patients treated^a^
Dimercaprol, 200 mg	Ampoule	27 356	800	5	20	1 368
Dimercaptosuccinic acid, 250 mg	Ampoule	6 500	750	5	15	433
Dimercaptosuccinic acid,100 mg	Capsule	11 000	300	15	45	245
Sodium calcium edetate, 500 mg	Ampoule	20 000	3 000	5	30	667
Succimer, 100 mg	Capsule	72 500	1 400	19	258	368
Succimer, 200 mg	Capsule	4 500	1 400	19	129	213
D-penicillamine, 250 mg	Capsule	1 000 000	1 000	15	60	16 666

^a^ Numbers of patients treated are based on numbers of doses requested by treatment centres: actual numbers treated were not recorded.

Note: Data were obtained from the Drug and Poison Information Centre, Food and Drug Administration of the Islamic Republic of Iran.

### Estimates of national prevalence

The exact number of patients in this ongoing outbreak is not known but, considering the high number of opium users in the country, many thousands are expected to be affected. According to an official report, 15 deaths in Tehran were confirmed due to lead poisoning from April to August 2016 (Iranian Legal Medicine Organization, unpublished data, September 2017). 

Using a reported rate of opium dependence of 1.62%^23^ in the Tehran population aged 15‒64 years of 5 million, we estimate there are 81 000 opium-dependent people in Tehran. Based on a report that 39.4% of 15‒64-year-olds in Tehran are regularly ingesting opium,^24^ we can estimate that 31 914 are oral opium users. From February 2016 to August 2017, 4294 patients were treated for lead poisoning at the two main referral hospitals in the city: 3794 at Loghman-Hakim hospital poison centre (Fig. 1) and almost 500 at another hospital (Behnoush B, Baharloo hospital, Tehran University of Medical Sciences, personal communication, September 2017). It is likely therefore that only about 13.5% of oral opium users in Tehran (4294/31 914) were treated for lead poisoning in the current outbreak. 

About 10% of the 80 million total Iranian population are living in Tehran and these two main referral centres serve 10% of the population of the country.^19^^,^^25^ We therefore estimate that 42 940 patients in the country were possibly treated for lead poisoning, of whom nearly 20 000 received lead-chelating agents (Table 1). Using a reported rate of opium dependence of 1.46% (in the whole country) and based on the 53 million Iranian population aged 15‒64 years,^2^ the estimated number of opium-dependent people is 773 800. Assuming again that 39.4% of them are oral users^24^ we estimate 304 887 oral opium users in this age group nationwide. As only about 43 000 users were treated by health professionals this suggests that there are more than 260 000 untreated oral opium users who may still be ingesting lead from contaminated opium supplies and may refer at a later date.

## Discussion

Investigating the cause of a toxicological outbreak requires careful epidemiological and toxicological analyses. In the decade since the first report was published,^26^ Iranian researchers have warned about high blood lead levels in opium-dependent patients.^11^^,^^18^^,^^21^^,^^26^ None of the studies, however, reported a mass outbreak of lead poisoning. After we completed our study, other poison treatment centres have published reports from the current outbreak.^15^^–^^17^

It has been suggested that lead is added to make the opium heavier.^18^^,^^21^ Adulteration of opium samples by soil, minced liver, flour, burned oil, tea, cacao, Indian henna, decoction of jute leaves, artificial leather, dried animal blood, melted X-ray films and pharmaceutical opioids, particularly tramadol, has been reported to make the opium heavier and gain more profit from sales.^27^^,^^28^ The additives used in the current outbreak are not known but the amount of lead in our analysis (3.55 mg in 1 g opium) is sufficient to poison an opium-dependent patient.^12^^,^^29^ According to a report released by the Iranian Drug Control Headquarters, testing of opium samples found at the Afghanistan border suggests that illegal opium sold in the Islamic Republic of Iran is adulterated inside our country.^26^ However, there is no official report confirming the deliberate addition of other substances to opium in the country. The ingestion lead can be detected by imaging techniques.^21^According to the World Health Organization, a tolerable weekly intake of lead is 25 µg/kg body weight (approximately 1750 µg for an average 70 kg adult).^30^ An opium user consuming 3–5 g of opium per day could have ingested around 10 500 to 17 500 µg of lead.^31^ Previous studies have reported that a median of 40 ppm (range: 5–37 000 ppm) of lead content in herbal products used in Indian Ayurvedic medicines might cause lead poisoning,^32^ suggesting that the potential lead content of the opium might cause acute poisoning in users. In conjunction with the high absorption rate of lead from the gastrointestinal tract,^30^ the amount of lead ingested (blood lead levels averaged 140.3 µg/dL in our sample) is high enough to cause serious lead intoxication.^31^^,^^33^ Although opium may be inhaled or ingested, a rapid situational assessment among dependent users in the country in 2007 showed that the oral route was the main route of consumption in 20.0% (1483/7425) of users of all types of substance and 39.6% (1194/3016) of opium users.^34^ Even those who normally smoke opium may sometimes ingest it. As the main route of lead toxicity is absorption from the gastrointestinal tract, a considerable proportion of Iranian opium users are therefore at risk of lead poisoning from opium.

We estimate that only about 13.5% of the 304 887 regular oral opium users nationwide have sought treatment and, although re-exposure is possible, more than 260 000 users are still at risk of lead poisoning. The estimated number of oral opium users was based on the study by the Iranian Drug Control Headquarters in 2011.^23^ Other studies, however, have estimated higher numbers of users. A large national study in 2013 estimated 1 728 000 ever-users of opium in the population,^31^ while the household mental health survey in 2011 estimated 1 181 900 opium users in the 15‒65-year-old Iranian population.^13^ Although none of those studies reported the route of consumption, these values are higher than our estimation of 773 800 oral opium-dependent people aged 15‒64 years nationwide. Therefore, our calculations might have underestimated the numbers of patients at risk of poisoning. Patients who are opium users may not present for treatment if there is stigma attached to opium use or if they only have mild symptoms. Also, not all patients treated may be recorded; this is particularly likely in outbreaks, when health-care providers face a high workload. Furthermore, patients may get treated at private clinics or remote hospitals. We based our estimations on available data for 15‒64-year-olds, however, opium use is popular among older age groups too in the country and they were not considered in our calculations. We assumed, based on previous evidence,^24^ that opium users are distributed around the country and not concentrated in the capital city.

Another limitation of our study is the lack of available data about the country’s opium supplies which could show the nature and extent of contamination. Data on lead contaminants in opium samples are limited due to the lack of available modern laboratories that are able to measure heavy metals in non-biological samples. The relation between blood lead level and factors such as dose, route of consumption, duration of use or age of users, were not studied. Although the total numbers of opioid medications distributed increased during the outbreak, we do not know if this is due to higher registration of opioid-dependent patients or to the higher demand for substitution therapy after withdrawal from contaminated opium.

This outbreak differs from other similar toxicological epidemics for several of reasons. First, the source of opioids is illegal and the quality of opium cannot be controlled. Other countries with a high prevalence of raw opium use may face similar challenges. Second, acute withdrawal syndrome after stopping opium use is a complication and patients need medical management to overcome the withdrawal syndrome, otherwise they will seek drugs from other illicit routes. Third, the availability of United States Food and Drug Administration-approved chelation therapy for lead positioning is limited in the Islamic Republic of Iran due to international sanctions that delay import of medications. Most low- and middle-income countries have no strategy for stocking chelation agents. Fourth, facilities for testing blood lead levels are not readily available in the majority of provinces in our country (and probably in most other low- and middle-income countries) causing a delay in starting effective therapy at emergency departments. Fifth, heroin is derived from opium and therefore the heroin sold in higher-income countries could be contaminated with high levels of lead if the heroin is derived from opium that has transited through the Iranian markets. Further studies might help to investigate this issue.

To prevent similar outbreaks, a surveillance system may be needed to monitor the illicit drug market and establish an early-warning network for contaminated supplies of drugs. This is important from the public health perspective, as well-established warning networks are able to observe any changes in illicit drugs, impurities, as well as substance abusers’ behaviour. Education is needed for health-care professionals and for the public, especially the large numbers of opioid abusers, about the risks of lead-contaminated opium. The illegal drug trade is a global chain that is not confined to one country and it should be followed from manufacturing to consumption everywhere in the world to monitor the risk of lead toxicity for opioid users.^11^^,^^21^ This approach could reduce the burden of disease particularly in the young populations who may be more vulnerable to substance use.^24^
